# Rosacea, microbiome and probiotics: the gut-skin axis

**DOI:** 10.3389/fmicb.2023.1323644

**Published:** 2024-01-08

**Authors:** Pedro Sánchez-Pellicer, Cristina Eguren-Michelena, Juan García-Gavín, Mar Llamas-Velasco, Laura Navarro-Moratalla, Eva Núñez-Delegido, Juan Agüera-Santos, Vicente Navarro-López

**Affiliations:** ^1^MiBioPath Research Group, Faculty of Medicine, Catholic University of Murcia (UCAM), Guadalupe, Spain; ^2^Eguren Dermatology and Aesthetics Clinic, Madrid, Spain; ^3^Gavín Dermatologists Clinic, Vigo, Spain; ^4^Department of Dermatology, Hospital Universitario de La Princesa, Madrid, Spain; ^5^Infectious Diseases Unit, Department of Internal Medicine, University Hospital of Vinalopó-Fisabio, Elche, Spain

**Keywords:** rosacea, microbiome, probiotic, gut microbiota, skin microbiota, gut-skin axis

## Abstract

Rosacea is an inflammatory skin disease involving diverse symptoms with a variable clinical progress which can severely impact the patient’s quality of life as well as their mental health. The pathophysiological model of rosacea involves an unbalanced immune system predisposed to excessive inflammation, in addition to vascular and nervous alterations, being certain cutaneous microorganisms’ triggers of the symptoms onset. The gut-skin axis explains a bidirectional interaction between skin and gut microbiota in some inflammatory skin diseases such as atopic dermatitis, psoriasis, or rosacea. The introduction and consolidation of the next-generation sequencing in recent years has provided unprecedented information about the microbiome. However, the characterization of the gut and skin microbiota and the impact of the gut-skin axis in patients with rosacea has been little explored, in contrast to other inflammatory skin diseases such as atopic dermatitis or psoriasis. Furthermore, the clinical evolution of patients with rosacea is not always adequate and it is common for them to present a sustained symptomatology with frequent flare-ups. In this context, probiotic supplementation could improve the clinical evolution of these patients as happens in other pathologies. Through this review we aim to establish and compile the basics and directions of current knowledge to understand the mechanisms by which the microbiome influences the pathogenesis of rosacea, and how modulation of the skin and gut microbiota could benefit these patients.

## 1 Introduction

Rosacea is a chronic skin disease affecting approximately 5.5% of general population, mainly patients between 45 and 60 years old, regardless of sex (Gether et al., 2018). Rosacea mainly appears in the cheeks, nose, chin, and forehead, with alternating periods of remission and aggravation. Cutaneous symptoms comprise persistent erythema, papules, pustules, telangiectasia, flushing, sebaceous glands hypertrophy, and fibrosis (characteristically referred to as phyma) (van Zuuren, 2017). In addition, more than 50% of rosacea patients present ocular rosacea even with absent or mild forms of cutaneous symptomatology. Symptoms and signs of ocular rosacea include dryness, burning, itching, photophobia, blurred vision, foreign body sensation, lid margin, conjunctival telangiectasia, meibomian glands collapse, and in severe forms can be developed corneal inflammation and perforation, scars, or vision loss (Redd and Seitzman, 2020).

Connecting with the chronic nature of rosacea, there is considerable evidence to support its association with various systemic comorbidities which could indicate a systemic inflammatory state. In this context, rosacea is linked with hypertension, dyslipidemia, atherosclerosis, other cardiovascular diseases, gastrointestinal diseases (this issue will be further developed), migraine, anxiety, depression, and even several malignancies (Morss-Walton and McGee, 2021).

The variable clinical spectrum mentioned above is illustrated by the multifactorial origin of rosacea. Therefore, several phenotypes of this diseases are recognized as part of an ongoing inflammatory process. On this basis, with current knowledge of its pathophysiology, the national rosacea society (NRS) published in 2017 a new standard classification system (Gallo et al., 2018). The previous 2002 classification yet identified the main signs and symptoms of rosacea and categorized them into four subtypes: erythematotelangiectatic (ETR), papulopustular (PPR), phymatous, and ocular rosacea, being ETR and PPR the top phenotypes diagnosed (Barakji et al., 2022). The problem with this system was that it did not consider the frequent coexistence of phenotypes and progression from one subtype to another. The updated 2017 classification requires at least one diagnostic criteria (fixed centrofacial erythema in a characteristic pattern that may periodically intensify, phymatous changes) or two major or primary phenotypes (papules and pustules, flushing, telangiectasia, ocular manifestations). In addition, secondary signs, and symptoms (burning, itching, edema, dryness) may also appear in conjunction with one or more diagnostic or primary phenotypes. The global rosacea consensus panel (ROSCO) recommendations supported in 2019 this NRS classification (Schaller et al., 2020). Importantly, rosacea can progress not only to additional phenotypes, but also in severity.

The treatment of rosacea should be based on these phenotypes (according to new NRS classification) (Gallo et al., 2018) and severity. Thus, there are several first-line treatments and in some cases a maintenance schedule could be justified depending on the clinical evolution and background of the patient. Usually in moderate to severe cases a combined approach of oral and topical therapy is required. Moreover, there are a variety of important general instructions for the management of several manifestations of rosacea, including non-aggressive hygiene measures, frequent use of moisturizers and photoprotectors, and elimination or mitigation of recognized aggravating factors (e.g., heat and some foods) (Schaller et al., 2017a; Salleras et al., 2019; Clanner-Engelshofen et al., 2022). However, rosacea is a difficult disease to keep under control. Some studies have shown that approximately 80% of rosacea patients consider their facial erythema to be unpredictable (Dirschka et al., 2015). In many cases the rosacea patient presents a history of therapeutic failure or insufficient results, but it should always be insisted for 6–8 weeks until an exacerbation treatment was considered ineffective (Schaller et al., 2017a; Salleras et al., 2019; Clanner-Engelshofen et al., 2022). At present, not all patients achieve complete resolution of symptoms. Therefore, there is still a need to find more effective treatments (van Zuuren et al., 2021).

A main reason for seeking new therapeutics approaches in rosacea is that its symptomatology can have an emotional impact and even on social relationships resulting in stigmatization. Numerous studies have highlighted a negative impact on the health-related quality of life in rosacea patients (van der Linden et al., 2015). Interestingly, in many patients the severity of rosacea does not correlate with psychosocial severity. Moderate cases already can have a severe psychosocial impact due to the facial location of the symptoms (Oussedik et al., 2018). Common psychosocial comorbidities of rosacea include depression and anxiety (Chang et al., 2022). In relation to associated mental health problems, in a descriptive study involving 827 European rosacea patients, one third reported feelings of stigmatization. These rosacea patients were more likely to avoid social situations (54.2% vs. 2.0%) and presented a higher rate of depression (36.7% vs. 21.1%) than patients without feelings of stigmatization (Halioua et al., 2017). In any case, stigmatization leads to even more difficulties, creating a vicious cycle. Moreover, and directly related to the psychosocial impact, almost half of patients with rosacea-associated facial erythema feel that it interferes with their work life (Bewley et al., 2016).

Finally, and focusing definitively on the objective of this review, it is mandatory to introduce the concept of the gut-skin axis and related promising therapeutic applications. The gut-skin axis explains how inflammatory skin diseases such as atopic dermatitis, psoriasis, acne vulgaris, hidradenitis suppurativa, or rosacea, are the consequences of a sophisticated interplay between genetic, lifestyle, and immune system in continuous synchronization with the nervous and endocrine systems (De Pessemier et al., 2021). Importantly, the cutaneous and gut microbiota play a key role in these relationships, as both skin and colon present constant interaction between microorganisms and the immune system (Salem et al., 2018). In addition, the introduction and consolidation of the next-generation sequencing (NGS) in recent years has enabled to obtain unprecedented information about the microbiome (Gilbert et al., 2018). Therefore, the aim of this narrative review is to analyze the role of gut and skin microbiota in the pathophysiology of rosacea (mainly in cutaneous rosacea), their composition and characteristics in these patients, and to overview the role of probiotics as a potential therapeutic target.

## 2 Pathophysiology of rosacea

**Figure 1:**
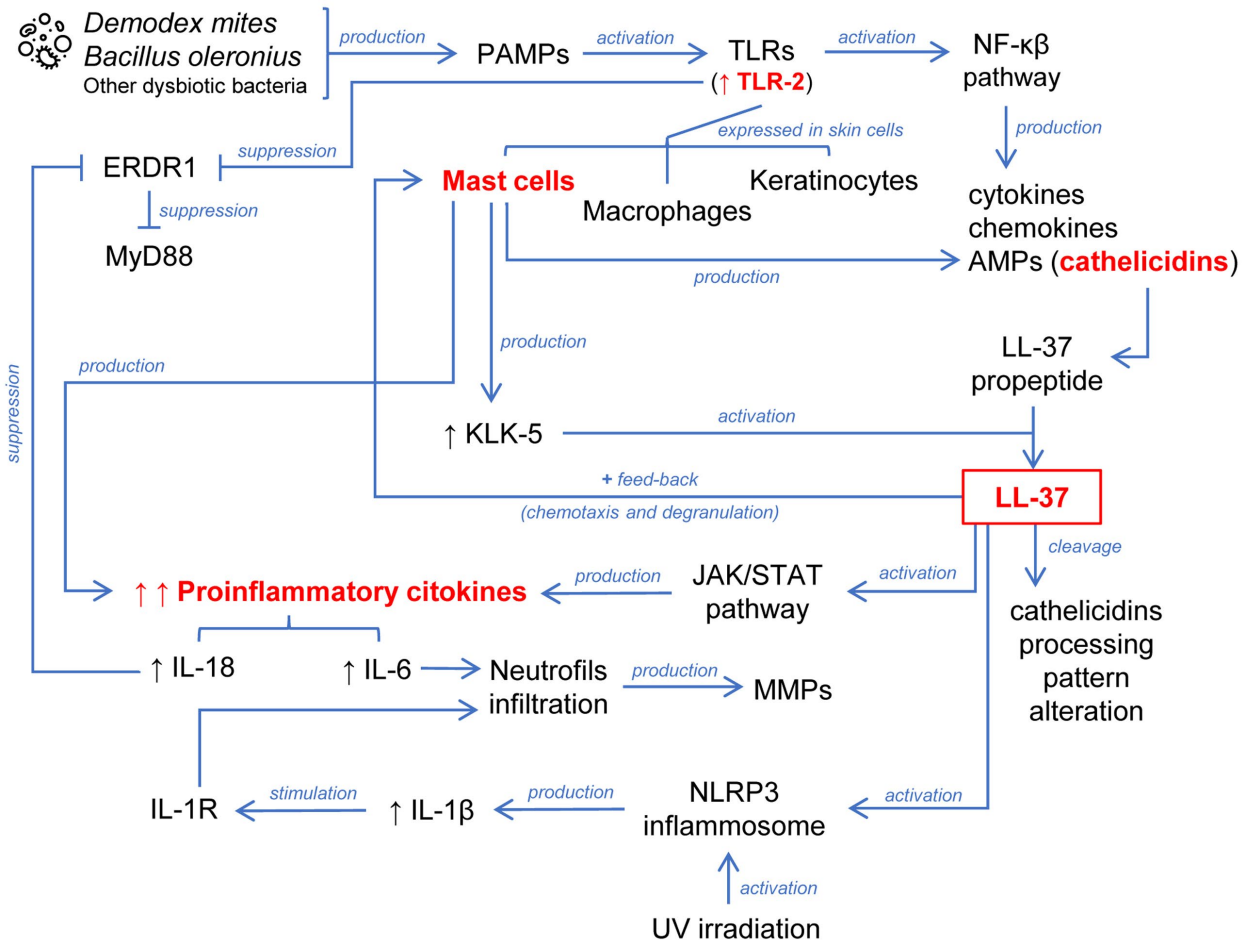
Schematic representation of the abnormally sustained innate immune response in the pathophysiology of rosacea. A key process is the activation of the cathelicidin LL-37 generated by different cells localized in the skin. This leads to chemotaxis and degranulation of mast cells through positive feedback. LL-37 is additionally involved in the inflammasome and JAK/STAT pathway activation. The release of proinflammatory cytokines converging these mechanisms leads to the infiltration of neutrophils.

**Figure 2:**
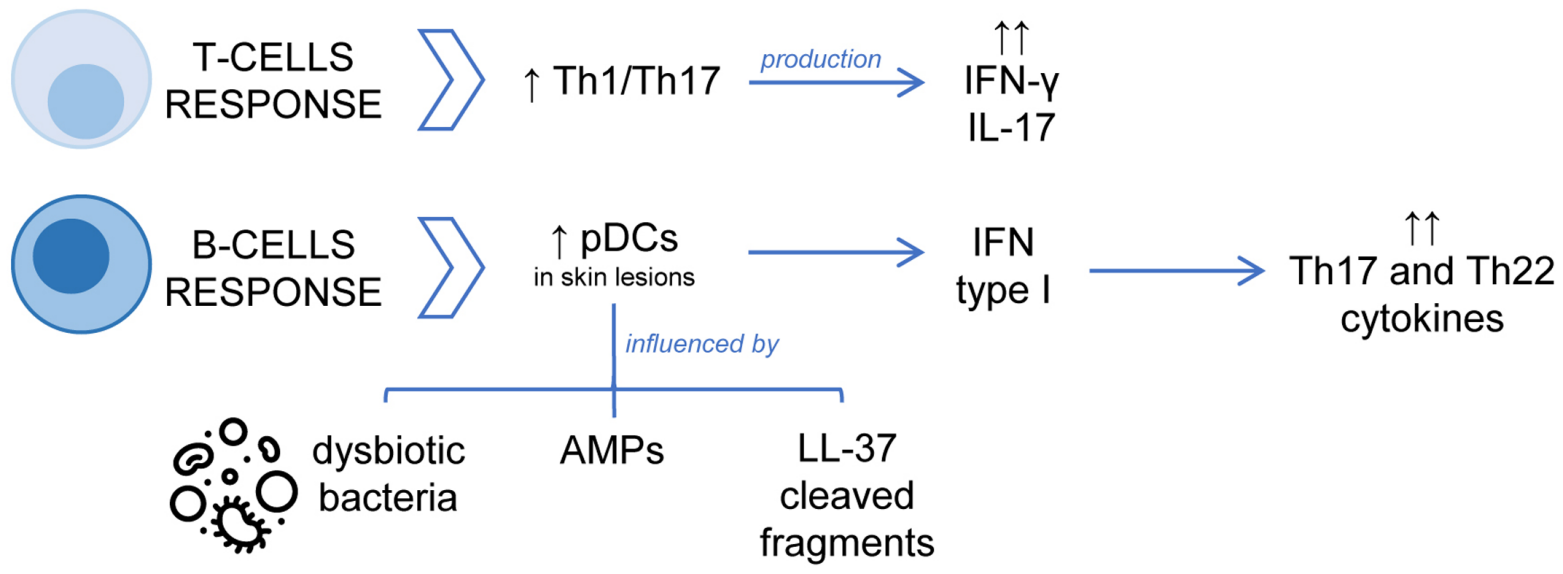
Schematic representation of the dysregulation of the adaptative immune response in the pathophysiology of rosacea. The T-cell response is dominated by Th1/Th17 cells, with significant increase of IFN-γ or IL-17. Concerning the B cell-mediated response, the accumulation and activation of pDCs with overexpression of type I IFN in skin lesions are important processes.

The pathophysiology of rosacea remains incompletely understood. The current rosacea pathophysiological approach suggests an unbalanced immune system predisposed to an excessive inflammation (Wladis and Adam, 2021) (Figures 1, 2), in conjunction with a vascular and neuronal dysfunction, and extrinsic or intrinsic triggers or exacerbating factors such as dysbiosis or several microorganism-related factors (Holmes, 2013; Chang and Huang, 2017), heat-cold, psychological stress, ultraviolet (UV) radiation, alcohol (Searle et al., 2021), smoking (Yuan and Yin, 2021), spicy food (Searle et al., 2021) among others (Wollina, 2019). Next, we will develop the main points of view on the pathophysiology of rosacea and its association with the microbiome.

### 2.1 Pathophysiology of rosacea: an immunological point of view

The pathophysiology of rosacea begins by the activation of cutaneous immune and nervous systems in response to physical, chemical, or biological stimuli. This subsequently leads to morphological changes observed in skin affected by rosacea such as facial erythema, telangiectasia, papules, or pustules. Activation of immune-mediated inflammatory pathways plays a central role in the pathogenesis of rosacea, involving several cell types, especially mast cells, and the release of certain proinflammatory mediators (Ahn and Huang, 2018; Marson and Baldwin, 2020; Wladis and Adam, 2021) (Figures 1, 2).

Pathogen-associated molecular patterns (PAMPs) derived from bacteria such as *Bacillus oleronius* (O’Reilly et al., 2012) or *Demodex* mites (Moran et al., 2017) are biological triggers which activate Toll-like receptors (TLRs) including TLR-2. TLRs play a key role in innate immunity and are expressed in keratinocytes, macrophages, and mast cells of the skin (Kumar, 2021). TLR stimulation leads to activation of the nuclear factor kappa B (NF-κB) pathway and consequent production of cytokines, chemokines, and antimicrobial peptides (AMPs). The problem begins when uncontrolled activation of the innate immune system implies a detrimental effect. Notably, TLR-2 is overexpressed in keratinocytes of rosacea patients (Yamasaki et al., 2011) enhancing skin sensitivity to external triggers because its stimulation activates an inflammatory cascade.

Cathelicidins are a class of AMPs stored in innate immune system and skin epithelial cells, which mediates leukocyte infiltration (Scheenstra et al., 2020), playing a major role in mammalian innate immune protection against bacteria. Moreover, cathelicidins are also overexpressed in the facial skin of rosacea patients (Yamasaki et al., 2007). Cathelicidin LL-37 (the only human cathelicidin) is secreted by keratinocytes like a biologically inactive propeptide form (called human cationic antibacterial protein of 18 KDa), requiring the action of kallikrein-5 (KLK-5) to release the biologically active peptide (Yamasaki et al., 2006). KLK-5 enzyme activity is enhanced in the skin of rosacea patients, which also explains the increased levels of LL-37 (Yamasaki et al., 2006, 2011). LL-37 could be further cleaved, which would affect its subsequent activity. In this regard, it has been observed that rosacea skin presents a different cathelicidin-processing pattern compared to healthy skin (Yamasaki et al., 2007).

Therefore, an exacerbated innate immune response is established in the skin of rosacea patients due to TLR-2 stimulation involving the production of the active form of the cathelicidin LL-37. In a healthy skin, activation of an innate immune response via TLRs would induce a controlled secretion of cytokines, chemokines, and AMPs, with recruitment and activation of leukocytes to eradicate the threat but without tissue damage. Rosacea patients do not experience the same balanced inflammatory response, so that there is a sustained anomalous innate immune response. In this regard, the role of mast cells in the pathogenesis of rosacea is remarkable (Wang et al., 2019). Mast cells are one of the major sources of cathelicidins and KLK-5 in the skin and are highly active in rosacea patients. In turn, released LL-37 exerts a powerful stimulus on the activity of mast cells inducing their chemotaxis, degranulation, and release of proinflammatory cytokines, generating a positive feedback mechanism. LL-37 has been injected intradermally into mast cell-deficient mice and no inflammation has been observed unlike in wild-type mice. However, when these mast cell-deficient mice have been supplied with mast cells and then injected with LL-37, they have exhibited inflammation (Muto et al., 2014). Moreover, inflammatory mediators secreted by LL-37-activated mast cells such as interleukin 6 (IL-6) lead to an infiltration of neutrophils that continue to amplify the feedback process releasing matrix metalloproteinases (MMPs) (Marson and Baldwin, 2020). KLK-5 can also be stimulated by MMP-9 in the skin of rosacea patients (Jang et al., 2011).

Mast cells also participate in the fibrosis processes observed in certain patients with phymatous rosacea. Histamine and tryptase secretion from mast cells can promote fibrosis by recruiting fibroblasts. Fibroblasts carry on stimulating mast cells causing a release of MMP-1, which can also influence more fibroblasts, facilitating fibrosis (Wang et al., 2019).

The Janus kinase/Signal transducers and activators of transcription (JAK/STAT) pathway has been also involved in the LL-37-mediated inflammatory mechanism of rosacea. Li et al. observed through an *in vitro* study with a keratinocyte cell line treated with LL-37, an increase in JAK2 and STAT3 activity in conjunction with an increase in the production of proinflammatory cytokines (Li et al., 2018).

The erythroid differentiation regulator 1 (ERDR1) is a recently identified cytokine widely expressed in many human tissues, localized in the inner part of the cytoplasmic membrane, and released through vesicles under stressful conditions (Houh et al., 2016). ERDR1 is negatively regulated by proinflammatory IL-18 and suppressed by both TLR-2 and the myeloid differentiation factor 88 (MyD88) pathways (Rodrigues-Braz et al., 2021). In this regard, Kim et al. evidenced that ERDR1 was reduced while IL-18 was increased in rosacea patients compared to healthy controls. These researchers employed a murine model to support the hypothesis of the participation of ERDR1 in the pathogenesis of rosacea. For this reason, they intradermally injected LL-37 into mice inducing the typical signs of rosacea but treatment with recombinant ERDR1 significantly reduced erythema and leukocyte infiltration (including CD4 and CD8 T-cells) (Kim M. et al., 2015).

The inflammasome is a caspase-1 activating multiprotein complex involving active IL-1β release and consequent stimulation of the interleukin 1 receptor (IL-1R) in many cells, with additional neutrophilic infiltration (Tang and Zhou, 2020). Recent studies have revealed that the nucleotide-binding oligomerization domain (NOD)-like receptors (NLR) family pyrin domain containing 3 (NLRP3) inflammasome activation plays a crucial role in LL-37-induced skin inflammation and rosacea pathogenesis (Yoon et al., 2021). Furthermore, it has been observed that UV irradiation increases inflammasome processing and a subsequent release of IL-1β. Thus, activation of the P2X purinoceptor 7 of keratinocytes by UV radiation and LL-37 enhances inflammasome activation. Therefore, LL-37 can modulate the proinflammatory effects of UV radiation contributing to increased susceptibility to sun exposure in rosacea patients (Salzer et al., 2014).

Recent studies have revealed the disruption of the mammalian target of rapamycin (mTOR) pathway in a variety of skin diseases (Karagianni et al., 2022). mTOR is a serine/threonine protein kinase involved in the coordination of a variety of signals regulating many fundamental cellular processes such as cell growth and differentiation, being crucial in skin homeostasis and shaping an appropriate epidermal barrier (Ding et al., 2016). Recently, Deng et al. reported that mTORC1 pathway is hyperactivated in rosacea (Deng et al., 2021). Transcriptional signatures and a cytoplasmic overexpression of the phosphorylated form of the S6 downstream molecule of mTORC1 in both epidermal and infiltrating cells, were found in facial biopsies from rosacea patients compared to healthy controls. Then, using a LL37-induced rosacea-like mouse model, both genetic ablation of mTORC1 and its pharmacological inhibition stopped the development of rosacea. The authors reported a positive correlation between epidermal activation of mTORC1 pathway and the severity of rosacea patients, revealing a mechanism linking dysregulation of the innate immune system and inflammatory response in this disease.

Adaptive immunity is also dysregulated in rosacea patients. The involvement of the adaptive immune system in the pathogenesis of rosacea is less well understood than relevance of the innate immune system. The T-cell response in rosacea is dominated by Th1/Th17 cells as evidenced by significantly increased interferon γ (IFN-γ) or IL-17. Macrophages and mast cells are increased in all subtypes of rosacea, whereas neutrophils reach a maximum in PPR (Buhl et al., 2015). Regarding B-cell-mediated response, Mylonas et al. have recently published that an overexpression of type I IFN in rosacea flare-ups correlates with the accumulation of plasmacytoid dendritic cells (pDCs) in the dermal infiltrate of skin lesions. In addition, this study showed that commensal skin bacteria are necessary for pDCs activation and type I IFN production, but in rosacea patients dysbiotic bacteria and AMPs increase this capacity. Moreover, cleaved fragments of LL-37 cause infiltration of pDCs into the skin, which are activated to produce high quantities of type I IFN inducing a strong immune response with increased expression of Th17/Th22 cytokines (Mylonas et al., 2023).

### 2.2 Pathophysiology of rosacea: a vascular and neurovascular point of view

Other important mechanism implicated in the pathogenesis of rosacea is the neurovascular hyperreactivity. An overexpression of some types of transient receptor potential (TRP) cation channels is found in several neuronal and non-neuronal cells of patients with rosacea (Sulk et al., 2012). The TRP channels are divided into several subfamilies (each subtype of TRP channels has different functions) and are localized in both sensory nerves and other non-neuronal cells such as mast cells, dendritic cells, endothelial cells, or keratinocytes, participating in nociceptive and neurogenic inflammatory processes (Rodrigues-Braz et al., 2021). Physical (temperature changes) or chemical stimuli (alcohol, spicy foods) activate these TRP channels triggering the secretion of vasoactive neuropeptides such as substance P (SP), vasoactive intestinal peptide (VIP) and calcitonin gen-related peptide (CGRP) (Marson and Baldwin, 2020). SP also induces mast cell degranulation expanding this neurogenic inflammation (Choi and Di Nardo, 2018). These mechanisms are related to the presence of telangiectasia and sustained flushing observed in rosacea patients in such a way that affected rosacea skin has a significantly lower threshold for heat and chemicals compared to non-affected skin.

Angiogenesis, persistent vascular and lymphatic dilation, and increased vascular permeability are also involved in the pathophysiology of rosacea. In skin biopsies of rosacea patients, the vascular endothelial growth factor (VEGF) expression is increased in epidermal and immune-infiltrating cells (Smith et al., 2007), being a critical regulator of angiogenesis. Moreover, LL-37 can increase VEGF levels in keratinocytes (Apte et al., 2019). In this regard, Chen et al. by means of a LL-37-induced rosacea-like murine model, showed that intraperitoneal thalidomide injection significantly alleviated erythema and reduced inflammatory cell infiltration, microvessel density and VEGF expression, in dermis (Chen et al., 2019). On the other hand, LL-37 can produce proangiogenic effects by activating the formyl peptide type 1 (FPR1) and the epidermal growth factor receptor (EGFR) in epithelial cells (Kajiya et al., 2017). It has also been reported an increased expression of the vascular cell adhesion molecule-1 (VCAM-1), the intracellular adhesion molecule-1 (ICAM-1) and E-Selectin in conjunction with high expression of LL-37, in facial skin biopsies of rosacea patients (Kulkarni et al., 2020). In addition, mast cell activation and recruitment on skin lesions as well promotes angiogenesis through the secretion of proangiogenic substances such as VEFG or fibroblast growth factor (FGF) (Shaik-Dasthagirisaheb et al., 2013).

### 2.3 Pathophysiology of rosacea: a genetic point of view

Some information indicates that there is a strong genetic predisposition and heritability of rosacea. Firstly, a family history is observed in more than one third of rosacea patients. Secondly, there is a high incidence of rosacea in certain populations such as Celtic and northern European descendants (Awosika and Oussedik, 2018). Furthermore, homozygous twins have higher NRS scores than heterozygous twins, and it has been estimated that the genetic contribution to NRS is close to 50% (Aldrich et al., 2015).

Moreover, an association of rosacea with some autoimmune diseases has been established (Egeberg et al., 2016). Interestingly, there are some shared associations between genes encoding human leukocyte antigen (HLA) variants and diseases such as celiac disease, type 1 diabetes, multiple sclerosis, inflammatory bowel disease, sarcoidosis, etc. (Awosika and Oussedik, 2018).

Nevertheless, the specific role of genetic in the development of rosacea is not fully elucidated, although in recent years several closely related associations with its pathophysiology have been evidenced. In this regard, Chang et al. conducted a genome-wide association study (GWAS) of 2,618 rosacea cases and 20,334 controls and identified one significant single-nucleotide polymorphism (SNP) associated with rosacea (Chang et al., 2015). This rosacea-associated intergenic SNP *rs763035* is positioned between *HLA-DRA* and *BTNL2* genes. Immunohistochemical skin analysis in PPR patients from this cohort reported the presence of HLA-DRA in epidermal Langerhans cells, BTNL2 in keratinocytes, and both in perifollicular infiltrates and endothelial cells. In addition, 3 HLA alleles such as HLA-DRB1*03:01, HLA-DQB1*02:01, and HLA-DQA1*05:01, were significantly associated with rosacea. These data could support the relevance of antigen presentation such as those from microorganisms in the pathophysiology of rosacea. Recently, Aponte et al. published other GWAS with 73,265 individuals of 97% European ancestry who self-reported rosacea (Aponte et al., 2018). Seven loci were identified including 2 related with skin and pigmentation phenotypes, 2 with inflammation 1 with both phenotypic categories, and 2 intergenic loci that were *a priori* unrelated to the pathophysiology of rosacea. Helfrich et al. in a case–control observational study of facial biopsies gene expression of ETR patients, revealed that some genes were overexpressed, being significantly remarkable those related with neuropeptides, mast cells and inflammation, matrix remodeling and AMP processing (Helfrich et al., 2015). Other case–control studies have identified some more SNPs in rosacea-associated genes providing evidence for the contribution of a genetic predisposition to the pathophysiology of rosacea (Yazici et al., 2006; van Steensel et al., 2008; Karpouzis et al., 2015; Akdogan et al., 2019; Hayran et al., 2019).

### 2.4 Pathophysiology of rosacea: a microbiological point of view

#### 2.4.1 Skin microbiota and pathophysiology of rosacea

Several microbes located on the skin have been associated with the pathogenesis of rosacea. The composition, stability and functionality of the cutaneous microbiota depends on the interactions between skin microorganisms and the conditions provided by the host (Byrd et al., 2018). The skin microbiota can prevent colonization by pathogens but in certain situations even beneficial or commensal bacteria can become pathogenic. This is noticed in many skin diseases (Sánchez-Pellicer et al., 2022) including rosacea. In this regard, Anna D. Holmes in 2013, based on these principles, established a multi-step model to explain the influence of the skin microbiota as a major player on the onset and progression of rosacea (Holmes, 2013). According to this model, firstly, hyperreactivity of TLRs or decreased tolerance to PAMPs from commensal or pathogenic bacteria, not activating under normal conditions, would trigger inflammatory pathways. This initial inflammation would modify the skin physiology and impact on the skin microbiota which will be reflected with the increased load of *Demodex* mites among other associations. This modification of the skin microbiota would affect the innate immunity which will exacerbate the whole inflammatory process contributing to the development of papulopustular lesions. Once a stable status has been reached between skin microbiota and innate immunity, a new imbalance would lead to the cyclical nature of rosacea. However, 10 years after the presentation of this model, knowledge of the pathophysiology of rosacea has increased, although it is not fully elucidated. In other words, it is not yet fully established whether these microorganisms are triggering factors of rosacea or whether they appear as a consequence of rosacea. This is a key issue still unresolved nowadays.

*Demodex* mites are recognized as commensal present in a diverse spectrum of host animals, being normal denizens of hair follicles and sebaceous glands because sebum is its main source of feeding. In contrast to other mites such as *Dermatophagoides*, they are both obligate commensals and host-specific. Characteristic *Demodex* species in humans are *Demodex folliculorum* and *Demodex brevis* (Foley et al., 2021). *Demodex* mites do not typically cause dermatological problems unless they reach a high load and/or penetrate the dermis. However, a strong association has been observed between density of *Demodex* mites and incidence of rosacea. A meta-analysis published in 2017 of case–control studies revealed that prevalence of *Demodex* colonization was 70.4% in rosacea patients vs. 31.8% in healthy controls, and the mean density was 71.0 mites/cm^2^ in rosacea patients vs. 8.7 mites/cm^2^ in healthy controls (Chang and Huang, 2017). Furthermore, in this meta-analysis, both ETR and PPR patients showed significantly higher *Demodex* density than healthy controls, although in PPR group tended to be greater than in ETR. The authors concluded that although this data cannot demonstrate a cause-effect relationship between *Demodex* mites and rosacea, there is an association suggesting mites could play a key pathogenic role. However, the authors stated as a limitation the great variability between studies at the level of different sampling, examination methods, and control groups.

Some research has shown that *Demodex* mites may itself contribute to the early inflammatory process in rosacea patients. On this issue, *Demodex* mites stimulate TLR-2 consequently increasing production of proinflammatory cytokines and playing a role in the continuum of rosacea pathogenesis (Lacey et al., 2018). In addition, mechanical blockage of the pilosebaceous unit due to *Demodex* overgrowth would also affect the skin barrier function and cause tissue damage (Moran et al., 2017). In contrast, there are some immune tolerance mechanisms that could explain a cutaneous proliferation of *Demodex* mites in rosacea patients. As we have mentioned previously, VEGF expression is increased in epidermal and immune-infiltrating cells such as lymphocytes, macrophages, and plasma cells (Smith et al., 2007). VEGF, due to its immunosuppressive properties, could induce T-cell proliferation and through collaboration with tolerogenic dendritic cells, promote the initial spread of *Demodex* mites (Forton, 2020) in a similar way that enhances the immune escape of tumor cells (Bourhis et al., 2021). Moreover, *Demodex* expresses the Thomsen-nouveau antigen (Tn Ag) (Kanitakis et al., 1997) which interacts with the macrophage galactose-type lectin (MGL) receptor of dendritic cells inducing their tolerance (Zaal et al., 2020). Thus, the inflammatory reaction could be insufficient to eradicate *Demodex* mites as some of the infiltrating T-cells have become dysfunctional, so that polymorphisms in dendritic cells would explain the different susceptibility to *Demodex* antigens (Forton, 2020). On the other hand, a recent meta-analysis evaluating the efficacy of different anti-*Demodex* treatments concluded that topical and systemic ivermectin, topical ivermectin-metronidazole, and topical tea tree oil, are promising anti-*Demodex* interventions (Li et al., 2023). In addition, topical ivermectin 1% treatment for 12 weeks significantly decreased *Demodex* density and downregulated IL-8, LL-37, TLR-4, human β-defensin 3 (HBD3), and tumor necrosis factor α (TNF-α) gene expression, implicating anti-inflammatory effects along improving clinical course of rosacea (Schaller et al., 2017b). Therefore, the role of *Demodex* mites in the pathophysiology of rosacea is complex and partially unknown, remaining unanswered questions about their immunostimulatory and immunotolerant activity.

*Bacillus oleronius* (the current name is *Heyndrickxia oleronia*) is a gram-negative bacterium that was first isolated from a *D. folliculorum* mite extracted from the face of a PPR patient. Remarkably, *B. oleronius* presented antigens that significantly stimulated the proliferation of peripheral blood mononuclear cells to a greater degree in rosacea patients than in controls (Lacey et al., 2007). Further studies have confirmed the immunoreactivity of rosacea patients to 62- and 83-kDa proteins of *B. oleronius* (O’Reilly et al., 2012; Jarmuda et al., 2014). Moreover, McMahon et al. demonstrated how *B. oleronius* proteins can induce neutrophil recruitment through activation of the inositol trisphosphate (IP3) pathway with production of proinflammatory cytokines (McMahon et al., 2016). Maher et al. showed that typical increased skin temperature of rosacea patients modified growth and protein pattern of *B. oleronius* leading to a greater production of immunoreactive proteins (Maher et al., 2018). Therefore, there is evidence that bacteria provided by *Demodex* mites can aggravate the established inflammatory response in rosacea. However, when Murillo et al. investigated the *Demodex* microbiota of rosacea patients by a culture-independent method did not identify *B. oleronius* in facial skin samples (Murillo et al., 2014a). Recently, Mylonas et al. found that *B. oleronius* amplifies type I IFN production by pDCs compared with other skin commensal bacteria. Nevertheless, the presence of *B. oleronius* was itself insufficient and a previous bacterial clearance by cathelicidin peptides was required, being specific microbial DNA the really trigger (Mylonas et al., 2023).

A recent study has revealed that *Corynebacterium kroppenstedtii* lives in mutualistic symbiosis with *D. folliculorum* and viable form of this bacterium seem to be an obligatory criterion for viability of the host (Clanner-Engelshofen et al., 2020). Rainer et al. found that *C. kroppenstedtii* was among the most abundant bacteria in rosacea subjects between 40 and 49 years, especially in patients with combined ETR and PPR, compared to an absence in their matched controls (Rainer et al., 2020). *C. kroppenstedtii* has been occasionally associated with human infections, mainly breast abscesses and granulomatous mastitis, but more studies will be necessary to clarify its real role in these diseases (Tauch et al., 2016). All these findings suggest that an effective antibiotic treatment against this bacterium could reduce the burden of *Demodex* mites and thus improve the clinical management of rosacea patients.

*Staphylococcus epidermidis*, a common skin inhabitant with beneficial effects (Byrd et al., 2018), was isolated in pure culture from pustular lesions of rosacea patients in contrast to the absence of growth from unaffected skin of these patients (Whitfeld et al., 2011). A few years earlier Dahl et al. published a study showing that *S. epidermidis* from facial skin of PPR patients secreted more proteins and generally more of each protein at 37°C compared with 30°C (Dahl et al., 2004). This data suggested that *S. epidermidis* would behave in a particular way due to changing skin conditions which are not present in patients not affected by rosacea, generating virulence factors. An extrapolation may be made to other bacteria which could modify its characteristics and contribute to the pathogenesis of rosacea under the conditions of a sick skin.

Given the similarity between acne vulgaris and some rosacea phenotypes, the role of *Cutibacterium acnes* in the pathophysiology of rosacea has been explored and suspected. *C. acnes* is a ubiquitous bacterium in healthy human skin being predominant in sebaceous regions, with a key role in cutaneous homeostasis, even acting in the prevention of pathogens colonization (Byrd et al., 2018; Sánchez-Pellicer et al., 2022). In general, *C. acnes* strains more associated with acne induce a more powerful inflammatory response than those less associated strains (Sánchez-Pellicer et al., 2022). Regarding rosacea, Jahns et al. investigated the presence of *C. acnes* in skin biopsies from PPR patients through an immunofluorescence assay (staining with a *C. acnes*-specific monoclonal antibody QUBPa3) (Jahns et al., 2012). In this study, skin biopsies from 82 rosacea patients and 25 controls were analyzed and only in 8.5% of rosacea patients were detected *C. acnes*. With these findings, the authors stated that *C. acnes* is unlikely to play a major role in the pathogenesis of rosacea. On the other hand, some researchers have hypothesized that removal of *C. acnes* from the skin microbiota is really what could contribute to the pathogenesis of rosacea (Marson et al., 2022). This is based on some recent studies using NGS of *16S rRNA* bacterial gene demonstrating a reduction of *C. acnes* compared to controls (Wang et al., 2020). However, Rainer et al. reported that *C. acnes* was the most representative bacterium in both rosacea patients and controls but was decreased in rosacea male patients compared to male controls (Rainer et al., 2020). Therefore, although *C. acnes* could apparently have a protective role against rosacea, some studies have reported contradictory results, and no causal relationship has been fully established. Some researchers have also proposed that like what occurs in acne (Sánchez-Pellicer et al., 2022), the specific pattern of *C. acnes* strains found in rosacea patients would be the fact particularly relevant in relation to rosacea pathophysiology (Thompson et al., 2021).

Systemic antibiotics have demonstrated efficacy in management of rosacea, specially in PPR patients (Xiao et al., 2023). However, the specific effect of these treatments on the cutaneous microbiota or on bacteria related to the pathophysiology of rosacea has barely been studied (Woo et al., 2020). There is a controversy in a practical order over the use of antimicrobial doses (50–200 mg) vs. anti-inflammatory doses (40 mg) of tetracyclines. However, several guidelines emphasize that it is not appropriate to use antimicrobial doses of antibiotics for the treatment of rosacea, as the administration of these drugs should only have an anti-inflammatory effect, and this effect is already obtained with doses as low as 40 mg of doxycycline (Salleras et al., 2019). This dose provides subantimicrobial levels of doxycycline, reducing the inflammatory response in rosacea patients without producing drug concentrations required to treat infections. Doxycycline can reduce the levels of reactive oxygen species (ROS) generated by neutrophils, can inhibit the expression of nitric oxide synthase, and can suppress the release of MMPs and proinflammatory cytokines (McKeage and Deeks, 2010).

#### 2.4.2 Gut microbiota and pathophysiology of rosacea

The human gastrointestinal tract contains over 100 trillion bacteria, the majority inhabiting the large intestine and forming the gut microbiota. These bacteria are involved in numerous metabolic reactions that substantially influence host physiology (Gilbert et al., 2018). The gut microbiota composition is mainly modulated to a greater degree by environmental or lifestyle factors such as diet or exposure to antibiotics. It also depends on age, sex, stress, diseases, and host-related genetic factors (Rothschild et al., 2018). The gut microbiota composition is stable in healthy adults and comprises a highly adaptive microbial community that constitutes a dynamic ecological balance (Lozupone et al., 2012). However, the gut microbiota could be exposed to disturbances altering this dynamic balance. A key characteristic of gut microbiota is the resilience so that there is a strong tendency to maintain its structure, meaning that it can continue being stable after a phase of modification and further recovery (Sommer et al., 2017). When this modifying factor is sustained and/or very powerful, so that it exceeds the resilience of the gut microbiota, this stable state disappears and the gut microbiota adapts to an alternative state, which could be dysbiotic.

The gut microbiota is closely involved with the immune system. Gut commensal bacteria act as regulators in the processes of immune tolerance (Takiishi et al., 2017). In fact, about 70% of lymphocytes are found in the gut-associated lymphoid tissue (GALT). Body areas colonized by microorganisms present the highest number of immune cells. Thus, changes in composition and diversity of the gut microbiota can lead to immunological and inflammatory disturbances in organs distant from the gut (Belkaid and Naik, 2013). In recent years, the concept of the skin-gut axis has been proposed to understand the pathophysiology of several skin chronic inflammatory diseases (O’Neill et al., 2016). The skin-gut axis describes how skin health is influenced by gastrointestinal health through the involvement of the immune system, metabolic-hormonal pathways, and the nervous system (Mahmud et al., 2022). The gut microbiota impacts on immunity which is recognized as the key regulator of the gut-skin axis and a gut dysbiosis impairs the balance of the immune system (Salem et al., 2018). While the exact mechanisms of the functionality of the gut-skin axis have not been fully established, there is growing evidence of the beneficial effects of probiotics in inflammatory skin diseases (Navarro-López et al., 2018, 2019; Sánchez-Pellicer et al., 2022) and this strikingly suggests that there is a complex relationship between the gut and the skin. Additional evidence for the relevance of the skin-gut axis is that many skin diseases co-exist together with non-cutaneous conditions such as gastrointestinal diseases (Wang and Chi, 2021; Thye et al., 2022).

The exact mechanisms of how the gut microbiota is linked to the onset and development of rosacea have not been fully established. By analogy with other immune-based inflammatory diseases such as atopic dermatitis, psoriasis, or acne vulgaris and with evidence of the existence of the gut–skin axis, it is likely that the main mechanism participates in the inflammatory immune response. A dysbiosis can lead to a compromised intestinal mucosal layer and impaired epithelial tight junctions, resulting in a worsening of the intestinal barrier function with translocation of bacteria and/or harmful compounds of bacterial origin (such as toxins or fragments of bacterial elements stimulating the immune system) from the gut into the bloodstream (Kinashi and Hase, 2021). A healthy gut microbiota maintains the integrity of the intestinal barrier by transforming complex polysaccharides into short-chain fatty acids (SCFA) (Koh et al., 2016). In addition, butyrate, a major SCFA, exerts a potent anti-inflammatory effect as it suppresses immune responses by inhibiting proliferation, migration, adhesion, and cytokine production by inflammatory cells (Salem et al., 2018). Enhanced PAMPs in the bloodstream and decreased butyrate of bacterial origin could also imply a hyperresponsiveness of B-cells and impaired differentiation of T-cells (Mahmud et al., 2022). Dysbiotic bacteria and/or harmful bacterial compounds along with altered immune cells, can reach the skin from bloodstream and impact on cutaneous physiology, pathology, and immune response (De Pessemier et al., 2021). As the pathophysiology of rosacea involves activation of the skin immune and nervous systems in response to physical, chemical, or biological triggers, these gut dysbiosis-driven changes could lead to disease progression, increased severity, flare-ups, or sustained symptomatology.

The coexistence of rosacea and gastrointestinal disorders has been documented. This supports the relationship between the gut and the skin in the pathophysiology of this disease. Egeberg et al. in 2016 published a Danish nationwide cohort study with 49,475 rosacea patients and 4,312,213 general population controls, investigating the association between rosacea and celiac disease, Crohn’s disease, ulcerative colitis, *Helicobacter pylori* infection, small intestinal bacterial overgrowth (SIBO), and irritable bowel syndrome (Egeberg et al., 2016). The baseline prevalence of all these gastrointestinal diseases was significantly higher in patients with rosacea compared to control subjects. However, through a 5-year follow-up survival analysis, adjusted hazard ratios did not reveal significant associations between rosacea and *H. pylori* infection and SIBO. Therefore, this large cohort study reported an increased prevalence of *H. pylori* infection and SIBO in patients with rosacea, whereas the risk of new onset of *H. pylori* infection and SIBO was not increased in rosacea patients. A singular question would be whether patients treated with antibiotics for SIBO or *H. pylori* infection will improve the symptomatology of rosacea. In this regard, a 3-year follow-up study evaluating the role of SIBO in the pathophysiology of rosacea revealed that SIBO treatment with rifaximin also led to clinical remission of rosacea in all patients, and then it persisted in the majority throughout follow-up period (Drago et al., 2016). Furthermore, this study revealed that the risk of SIBO is significantly higher in PPR than in ETR. Remission of rosacea concomitant to SIBO treatment has been evidenced in other studies (Wang and Chi, 2021). The subjacent mechanism relating SIBO to rosacea has not been clarified. Nevertheless, bacterial invasion in the small intestine leads several pathological consequences such as direct mucosal injury, toxins, malabsorption, decreased brush border enzyme activity, excessive H_2_ and CH_4_, among others (Bushyhead and Quigley, 2022). On the other hand, Jørgensen et al. published in 2017 a meta-analysis comprising 928 rosacea patients and 1,527 controls, highlighting a significant association between *H. pylori* infection only if the diagnostic was restricted to breath test. In addition, the effect of eradication treatment on rosacea symptoms was not significant (Jørgensen et al., 2017). Unlike SIBO, a connection between *H. pylori* and rosacea is apparently better established. *H. pylori* infection can trigger a cytotoxic reaction inducing release of TNF-α and IL-8 due to the factor virulence cytotoxin-associated gene A (CagA), aggravating the inflammatory reaction involved in the pathophysiology of rosacea. Moreover, *H. pylori* can impact on skin conditions by increasing N_2_O concentration leading to vasodilatation and inflammation (Yang, 2018).

## 3 Analysis of the skin microbiota in rosacea patients

Studies characterizing the skin microbiota in rosacea patients are scarce and relatively recent (Table 1). New culture-independent techniques based on NGS of the *16S rRNA* gene have allowed an increasingly comprehensive characterization of the microbiome (Gilbert et al., 2018). In the past, skin microorganisms were characterized by culture methods, but these underestimated the complete diversity of the cutaneous microbiota. Therefore, to overcome the limitations of microbiological culture and to understand the full diversity of the skin microbiota, sequencing methods have been applied. Let us review the research on the topic to date.

**Table 1:** Main studies including skin microbiota data in rosacea patients.

Study	Methodology and study population	Key results
Zaidi et al. (2018)	60 twins (32 without rosacea, 18 with rosacea, 32 matched) Affected (cases) an unaffected (controls) skin from cheeks sampled using Sebutape^®^ strips	No significant difference was observed regarding α-diversity between rosacea and non-rosacea monozygotic twin pairs PCoA did not demonstrate a separate clustering between rosacea patients and healthy controls No significant difference was observed in relative abundance of any predominant phylum between rosacea patients and healthy controls *Gordonia* and *Geobacillus* genera and age range “30–60 years” were significantly predictive of NRS score
Rainer et al. (2020)	Case–control study 19 individuals with ETR, PPR or both, and 19 age- and sex-matched controls Skin from nose and cheeks using sterile swabs	Greater richness in rosacea patients vs. paired controls without statistically significant degree Analysis of similarity did not show clustering between ETR and PPR patients and matched controls *Cutibacterium acnes*, majority species in both rosacea patients and controls *Corynebacterium kroppenstedtii* increased in rosacea patients mainly with combined ETR and PPR
Thompson et al. (2020)	Data analysis extension of Rainer 2020 study	Significant increase in *Campylobacter ureolyticus* and *Prevotella intermedia* and a depletion in *Acinetobacter* PPR patients compared to controls
Thompson et al. (2021)	Case–control study 19 individuals with ETR, PPR or both, and 19 age- and sex-matched controls (same rosacea cohort as Rainier 2020 study) 8 acne patients and 8 age- and sex-matched controls. Skin from nose and cheeks using sterile swabs	Significantly higher α-diversity in acne patients than in rosacea patients No significant difference was observed regarding α-diversity between cases and controls of both conditions Different clustering in PCoA plot between cases and controls for acne and cases and controls for rosacea Proteobacteria, majority phylum in acne patients Actinobacteria, majority phylum in rosacea patients *Serratia marcescens* and *Cutibacterium acnes* were increased in rosacea patients vs. acne patients *Cutibacterium acnes* abundance relative in PPR patients was like that in acne patients
Woo et al. (2020)	Rosacea patients with IGA 3 and 4 Skin from cheeks using sterile swabs Skin microbiota analysis before and after 6 weeks doxycycline oral treatment	α-diversity did not change regarding age (older and younger than 60 years), IGA, before and after doxycycline treatment Weak clustering non-significant based on analysis of similarities both per patient and per treatment *Weissella confusa* increased significantly after doxycycline treatment *Cutibacterium acnes* presented a significantly higher relative abundance in IGA 3 patients *Snodgrassella alvi* presented a significantly higher relative abundance in IGA 4 patients
Wang et al. (2020)	Case–control study 21 ETR, 15 PPR and 22 healthy controls Skin fungal and bacterial communities’ analysis Skin from cheeks using sterile swabs	α-diversity increased in PPR patients compared to healthy controls ETR patients presented a higher relative abundance of Firmicutes phylum compared to healthy controls PPR patients presented a lower relative abundance of Actinobacteria phylum compared to healthy controls *Cutibacterium* was significantly decreased in both ETR and PPR patients *Staphylococcus* was increased in ETR patients *Streptococcus* was increased PPR patients Significant changes were not observed in the rosacea-associated fungal microbiome PCoA did not demonstrate a separate clustering of fungal microbiome and bacterial microbiota between rosacea patients and healthy controls
Murillo et al. (2014a)	*Demodex* microbiota 15 ETR, 15 PPR and 17 sex and age-matched healthy controls Skin biopsies of alar crease	Actinobacteria, dominant phylum in ETR patients and controls but greatly diminished in PPR patients Proteobacteria and Firmicutes, increased in PPR patients *Duganella zoogloeoides*, the most represented species in ETR patients *Acinetobacter pitii*, the most represented species in PPR patients Pathogens such as *Bartonella*, Haemophilus or *Escherichia* were only observed in rosacea patients

Zaidi et al. published in 2018 a study involving 60 twins over 18 years of age (mainly monozygotic), of which 18 presented rosacea (32 participants matched) (Zaidi et al., 2018). The authors analyzed the skin microbiota from bilateral malar cheeks using Sebutape® strips (affected skin for cases and unaffected skin for controls). This twin design provided genetic, and in many cases environmental, control and an appropriate matching. No significant difference was detected in the α-diversity Shannon index between rosacea affected and non-rosacea affected monozygotic twin pairs. Then, the authors performed a correlation analysis between the NRS score and the Shannon index of matched twins, showing a negative association and suggesting that rosacea severity negatively affects bacterial diversity, but without statistical significance. A principal coordinate analysis (PCoA) based on weighted and unweighted UniFrac or Bray-Curtis distances did not demonstrate a significant separate clustering between rosacea patients and healthy controls, although monozygotic twins presented a more similar skin microbiota than dizygotic twins. Furthermore, no significant difference was observed in the relative abundance of the predominant phylum in subjects with and without rosacea, which were in descending sequence Firmicutes, Proteobacteria, Actinobacteria, and Bacteroidetes. Interestingly, a univariate random effect Poisson regression (REPR) showed a significant association between NRS score and *Gordonia*, *Blautia*, *Chryseobacterium*, *Wautersiella* and *Geobacillus* genera. Multivariate REPR revealed that *Gordonia* and *Geobacillus* and age range 30–60 years were significantly predictive of NRS score. These results support the hypothesis that rosacea severity is related and linked to skin microbiota.

Rainer et al. presented in 2020 a case–control study with 19 adult subjects diagnosed with ETR, PPR, or both, age and sex-matched with 19 controls without rosacea (Rainer et al., 2020). These researchers evaluated the cutaneous microbiota of the nose and cheeks using sterile foam-tipped swabs. Skin microbiota with greater richness was evidenced in rosacea patients vs. paired controls by means of a phylogenetic diversity whole tree metric obtained from rarefaction curves, but without statistically significant degree. Analysis of similarity using weighted UniFrac distances did not show any clustering between ETR and PPR skin samples vs. their matched controls. Concerning the abundance of majority species, *C. acnes* was the most representative in both rosacea patients and controls. However, although *C. acnes* was increased in male controls (57%) compared to female controls (30%), the relative abundance was similar between male (24%) and female (28%) rosacea patients. *C. kroppenstedtii* was the second most abundant species in rosacea patients, reaching 6% in subjects between 40 and 49 years as compared to the virtual absence in their matched controls. Among the different rosacea subtypes, *C. kroppenstedtii* was highly increased in patients with combined ETR and PPR (19%) and was 5 and 1% in patients with PPR and ETR, respectively. Therefore, only remarkable differences between different rosacea subtypes and controls were detected at species-specific level. Thompson et al. published also in 2020 an extension of the results of this study highlighting other interesting species-level differences between study groups (Thompson et al., 2020). It was emphasized a significant increase in *Campylobacter ureolyticus* and *Prevotella intermedia* and a depletion in *Acinetobacter* in the skin microbiota of PPR patients compared to controls. These associations could be responsible for a relationship between rosacea and its comorbidities (Haber and El Gemayel, 2018).

This last researcher group published another recent microbiome study in acne and rosacea patients (Thompson et al., 2021). These skin conditions follow different clinical courses with some similar clinical manifestations, suggesting there are fundamental pathophysiological differences. The authors considered whether the skin microbiota (samples from bilateral cheeks and nose) could explain such differences and conducted a case–control study with 8 acne patients matched to 8 controls and 19 rosacea patients matched to 19 controls [same rosacea cohort as (Rainer et al., 2020)]. Notably, acne population was younger and more racially diverse than rosacea population. The Shannon index revealed significantly higher α-diversity in acne patients than in rosacea patients. However, α-diversity was similar between cases and controls for both conditions. Using a weighted UniFrac distance analysis, a significant difference was observed between all study groups, but even when examining at the PCoA plots, clustering was apparent between cases and controls for acne and cases and controls for rosacea. Proteobacteria was the most abundant phylum in acne patients and was significantly increased compared to rosacea patients. Actinobacteria was the most abundant phylum in rosacea patients and was significantly increased compared to acne patients. *Serratia marcescens* and *C. acnes* were both increased in patients with rosacea vs. patients with acne. The authors suggested that the different relative abundance of *C. acnes* in the study groups could reflect a specific pattern of *C. acnes* strains. For example, the relative abundance of *C. acnes* in rosacea patients with inflammatory papules and pustules was comparable to that in acne patients, but lower than in rosacea patients without inflammatory papules and pustules. This is of interest as rosacea with inflammatory papules and pustules are clinically close to acne.

By means of a different approach regarding these studies, Woo et al. reported in 2020 a study with 12 rosacea patients with severity scores 3 and 4 using the investigator’s global assessment (IGA) grading scale (Woo et al., 2020). The skin microbiota from their cheek before and after an oral treatment with doxycycline for 6 weeks was examined. No changes in α-diversity indices were observed regarding age (older and younger than 60 years) or rosacea severity before and after treatment with doxycycline. Analysis of similarities based on weighted UniFrac distance evidenced a weak clustering of samples per patient and per treatment (both not significant) suggesting interindividual variability of the skin microbiota and an associated resilience. The most abundant species on the skin of rosacea patients before treatment with doxycycline were *Staphylococcus epidermidis* (28%), *C. acnes* (13%), *Pseudomonas koreensis* (8%), *Actinetobacter haemolyticus* (7%) and *Snodgrassella alvi* (6%). The most abundant species on the skin of rosacea patients after treatment with doxycycline were *S. epidermidis* (22%), *Stenotrophomonas rhizophila* (8%), *C. acnes* (7%) and *Corynebacterium tuberculostearicum* (7%). Relative abundance of *Weissella confusa* increased significantly after doxycycline treatment. Regarding severity, *C. acnes* exhibited a significantly higher relative abundance in IGA 3 patients while *S. alvi* in IGA 4 patients. This study showed that the cutaneous microbiota in rosacea patients had some specific characteristics depending on age and severity and, importantly, is modified by a systemic antibiotic treatment.

Another case–control study was that of Wang et al. characterizing the cutaneous fungal community in rosacea patients in addition to the cutaneous bacterial ecosystem (Wang et al., 2020). Twenty-one ETR patients, 15 PPR patients, and 22 healthy subjects (50 women and 8 men between 18 and 64 years) were included. Skin swabs were collected from both cheeks for sequencing and analysis of *16S rRNA* and *ITS1* amplicons. Regarding bacterial microbiota, ETR and PPR patients presented a higher and lower relative abundance of Firmicutes and Actinobacteria compared to healthy controls, respectively. *Cutibacterium* was the dominant genus in healthy controls and its relative abundance was significantly decreased in patients with ETR and PPR. *Staphylococcus* and *Streptococcus* showed different behavior depending on the rosacea subtype, with *Staphylococcus* increasing in patients with ETR and *Streptococcus* increasing in patients with PPR. However, no significant difference was observed based on mild, moderate, or severe forms of rosacea. Moreover, no significant changes were observed in the fungal microbiome associated with rosacea, being dominant *Malassezia* and *Alternaria* genera. Likewise, an increase in the Shannon index was observed in PPR patients compared to healthy controls, but no difference was evidenced at mycobiome level. PCoA based on weighted UniFrac distance of bacterial and fungal microbiome also did not demonstrate a different clustering between rosacea patients and controls.

As mentioned above, subjects suffering rosacea present a significantly higher prevalence of the degree of *Demodex* mite infestation and this could play a role in the rosacea pathophysiology. In this way, Murillo et al. characterized the specific microbiota of *Demodex* mites in 15 ERT subjects, 15 PPR subjects and 17 sex and age-matched healthy controls (Murillo et al., 2014a). Notably, this was the first study using a culture-independent method (*16S rRNA* sequencing) for analysis of the microbiota of *Demodex* mites from skin biopsies (alar crease). Phylum composition was reported to be significantly different in PPR patients compared to ETR patients and healthy controls. Actinobacteria was the dominant phylum in ETR patients and controls but was greatly diminished in PPR patients. In addition, Proteobacteria and Firmicutes increased in PPR patients compared to ETR patients and controls. *C. acnes*, *S. epidermidis*, *C. kroppenstedtii*, *Streptococcus mitis*, *Propionibacterium granulosum* and *S. alvi* were the 6 species shared by the 3 study groups. *Duganella zoogloeoides* and *Acinetobacter pitii* were the most represented species in the *Demodex*-specific microbiota of ETR and PPR patients, respectively. Interestingly, pathogens such as *Bartonella*, *Haemophilus* or *Escherichia* were only observed in rosacea patients. In fact, *Bartonella quintana* which is known to causes trench fever and chronic bacteremia, endocarditis, and bacillary angiomatosis, was detected in one subject of this rosacea cohort (Murillo et al., 2014b). The authors concluded that the mite microbiota in rosacea patients could differ according to the host status, although only a limited number of mites were analyzed in healthy controls compared to rosacea patients due to a lower density of *Demodex*.

## 4 Analysis of the gut microbiota in rosacea patients

Studies characterizing the gut microbiota in rosacea patients are also very scarce, very recent, with small sample sizes and all based on NGS of the *16S rRNA* gene. Therefore, conclusions are particularly difficult to achieve. According to the current knowledge of the gut-skin axis and considering the potential relevance of modulating the intestinal microbiota as a therapeutic target for rosacea, it is imperative that further research will be conduct in this area.

Nam et al. reported in 2018 a study to establish relationships between the gut microbiota of 12 rosacea patients (50% ETR, 17% PPR and the remainder were of unknown subtype) and 251 healthy controls, all females (Nam et al., 2018). These researchers did not find significant differences at α- and β-diversity level between rosacea and rosacea-free groups. However, significant differences were identified at genera level. *Acidaminococcus* and *Megasphaera* were more abundant and Peptococcaceae family unknown genus and *Methanobrevibacter* were relatively lacking in rosacea subjects compared to healthy controls. Furthermore, after an adjustment by some variables (age, body mass index, diabetes type 2, gastric polyps, colon cancer) *Acidaminococcus*, *Megasphaera*, and Lactobacillales were significantly increased in rosacea patients compared to rosacea-free subjects, as Peptococcaceae, *Methanobrevibacter*, *Slackia*, *Coprobacillus*, *Citrobacter*, and *Desulfovibrio* were significantly decreased.

Chen et al. in 2021, published another similar study although obtaining quite different results (Chen et al., 2021). They compared the gut microbiota of 11 patients with rosacea and 110 age- and sex-matched healthy controls. Most of the rosacea patients were female, but mean age (53 years) and ETR patients (50%) was higher than in Nam 2018 study (Nam et al., 2018). A decrease in richness but not in α-diversity was observed in rosacea patients. The PCoA based on the unweighted UniFrac distance showed a different clustering between both study groups, suggesting a dissimilar gut microbial structure. In addition, the inclusion of covariates such as alcohol, tea or yogurt consumption, tobacco, exercise, vegetarianism, or rosacea subtype did not affect the profile of the gut microbiota structure in PCoA. Although both groups presented a gut microbiota dominated by Bacteroidetes, Firmicutes, and Proteobacteria, rosacea patients presented a higher abundance of *Bacteroides* and *Fusobacterium*, and a lower abundance of *Prevotella* and *Sutterella* than the controls. Using a linear discriminant analysis effect size (LEfSe), increases of *Rhabdochlamydia*, *Bifidodbacterium*, *Sarcina*, and *Ruminococcus*, and decreases of *Lactobacillus*, *Megasphaera*, *Acidaminococcus*, *Haemophilus*, *Roseburia*, *Clostridium*, and *Citrobacter,* were identified as characteristics of rosacea patients. The authors went one step further and investigated the functional profile of the samples through a phylogenetic investigation of communities by reconstruction of unobserved states (PICRUSt) analysis. Gene families implicated in arylsulfatase A related enzymes, glycosiltransferease, and cobalamin transport were more abundant in rosacea patients. However, ABC-type sugar and amino acid transport system related enzymes, chemotaxis and transcription related enzymes were less abundant in rosacea patients.

The most recently published study is that of Moreno-Arrones et al. evaluating the gut microbiota of 15 PPR patients (mean age 36 years, 80% females) and 15 controls (mean age 39 years, 33% females) (Moreno-Arrones et al., 2021). The CHAO1 richness index was increased in PPR patients. A canonical correspondence analysis (CCA) showed a significant different clustering between cases and controls. By means of a LEfSe analysis was identified a decrease of *Prevotella copri*. Moreover, an increase of Bacteroidales order, Syntrophomonadaceae and Lachnospiraceae families, *Anaerovorax* and *Tyzzerella* genera, and *Akkermansia muciniphila* and *Parabacteroides distasonis* species, was established as compositionally characteristic of PPR status.

## 5 Probiotics as a therapeutic target in rosacea patients

The modulation of the skin and gut microbiota due to its potential influence on the pathogenesis of rosacea could be an interesting therapeutic target. As the international scientific association for probiotics and prebiotics (ISAPP) stated, probiotics are live microorganisms that, when administered in adequate amounts, confer a health benefit on the host (Hill et al., 2014). Randomized clinical trials have shown beneficial results in the clinical course of inflammatory skin diseases such as atopic dermatitis (Navarro-López et al., 2018), psoriasis (Navarro-López et al., 2019), or acne vulgaris (Jung et al., 2013). However, there is a general lack of clinical and preclinical evidence regarding probiotics and rosacea. The clinical development of symptoms of rosacea patients, despite the current therapeutic arsenal, is not always appropriate and many times these patients show a maintained symptomatology with frequent relapses. There is still an ongoing need for more efficacious treatments (van Zuuren et al., 2021).

Manzhalii et al. conducted an open-label, randomized clinical trial in 57 patients with erythema and papulopustular lesions, of which 36% were patients with PPR (the remaining 22 and 57% were, respectively, diagnosed with acne and seborrheic dermatitis) (Manzhalii et al., 2016). The patients were divided into 2 groups and one of them was treated with standard topical therapy consisting of tetracyclines, corticosteroids and retinoids. The other group was treated with the same topical therapy plus oral administration of the probiotic strain *Escherichia coli* Nissle 1917. After 1 month of follow-up, 32% patients of probiotic group showed recovery and 57% significant amelioration, compared to 17% patients of control group showing recovery and 39% significant amelioration. Furthermore, patients treated with the probiotic evidenced an increase in the quality-of-life questionnaire score. Post-treatment stool culture indicated that therapy with *E. coli* Nissle 1917 caused an increased growth of *Lactobacillus* and *Bifidobacterium*, and a reduction of *Staphylococcus*, yeasts, *Bacteroides*, *Proteus*, *Citrobacter*, and *Klebsiella*. Therefore, Nissle strain improved the clinical progression of these patients with a substantial modification of the gut microbiota.

Buianova et al. published in 2018 other randomized clinical trial with 60 rosacea patients as a short communication (Buianova et al., 2018). These study subjects were separated in 2 groups. Thirty rosacea patients were treated 1 week with oral antibiotics, vitamins, antihistamine, and topical permethrin. The remaining 30 rosacea patients were added a mixture containing a *Bifidobacterium* strain 5 × 10^7^ CFU 3 times per day and polyoxidonium (immunomodulator) for 3 weeks. In the probiotic group 57% of patients experienced complete clinical remission compared to 28% in the control group. In addition, a stool culture at the end of the treatment period indicated an increase in *Lactobacillus* and *Bifidobacterium* burden in rosacea patients of the probiotic group.

A case report illustrated the efficacy of an oral antibiotic and probiotic combined therapy in a patient with scalp rosacea (Fortuna et al., 2016). This patient presented papules and pustules located on face and scalp with an intermittent erythema and burning sensation along with blepharitis and conjunctivitis. The patient was treated for 8 weeks with 40 mg of doxycycline per day and a probiotic mixture composed of *Bifidobacterium breve* BR03 *and Lactobacillus salivarius* LS01 10^9^ CFU 2 times per day. After 8 weeks, antibiotic treatment was stopped but the probiotic continued. The patient significantly improved both cutaneous and ocular symptoms and after 6 months did not present any relapse.

Another therapeutic option beyond oral probiotics are topical probiotics. Topical application of probiotic bacteria could improve the natural barrier of the skin by exerting a direct effect at the site of application. However, in general, there have been few clinical trials evaluating the efficacy of topically applied probiotics (Habeebuddin et al., 2022). Regarding rosacea, a recent clinical trial has explored the efficacy of the product M89PF containing Vichy volcanic mineralizing water, probiotic fractions of *Vitreoscilla filiformis*, hyaluronic acid, niacinamide, and tocopherol (Berardesca et al., 2023). *V. filiformis* extract topically applied presents several interesting properties such as optimizing cell immunity, protecting against pathogen skin bacteria, and improving skin barrier function (Gueniche et al., 2021). In this clinical trial, 20 rosacea patients were randomly assigned to receive M89FP or non-medical cosmetic standard skin care over every half-face side for 30 days. M89FP therapy significantly enhanced skin hydration as reduction of the transepidermal water loss (TEWL), decreased *Demodex* density, improved erythema (measured by chromameter), and improved self-perception of skin erythema, tightness, and dryness.

Some studies have found associations between certain bacterial skin colonization profiles and impairment of cutaneous barrier function specifically in patients with rosacea (Yuan et al., 2020). Therefore, an altered skin barrier could promote the overgrowth of key bacteria on the skin aggravating the symptomatology of rosacea. In this regard, probiotics contributing to restore the natural skin barrier could improve the clinical course of rosacea. Let us review some probiotic strains with beneficial effects on the skin barrier function. *Lactobacillus paracasei* CNCM-I 2116 was able to induce a faster function barrier recovery after impairment with sodium lauryl sulfate using an *ex vivo* skin organ culture (Gueniche et al., 2010). In addition, 3 weeks of high doses of this strain significantly reduced the TEWL in a murine model of sensitized skin with dinitrochlorobenzene (Philippe et al., 2011). A randomized, double-blind, placebo-controlled, clinical trial also demonstrated that supplementation for 2 months with this strain decreased skin sensitivity and increased skin barrier recovery (Gueniche et al., 2014). In a reconstructed human epidermis model, a lysate of *Lactobacillus reuteri* DSM 17938 enhanced laminin A/B levels which are important extracellular matrix proteins, suggesting a beneficial effect on skin barrier (Khmaladze et al., 2019). A randomized, double-blind, placebo-controlled clinical trial demonstrated that oral intake of *Lactobacillus plantarum* HY7714 at 10^10^ CFU per day for 12 weeks, suppressed the TEWL in facial and forearm skin along with an increase in skin water content (Lee et al., 2015). The same research group demonstrated in an observational study that healthy volunteers who received this probiotic strain developed changes in their gut microbiota with an increase of *Bifidobacterium* and a decrease of Proteobacteria, along with a decrease in MMP-2, MMP-9, zonulin, and calprotectin plasma levels, all of which are related to skin and intestinal permeability (Nam et al., 2020). In addition, RNA-seq analysis showed increased expression of genes related to the integrity of the intestinal barrier. Furthermore, oral treatment with *L. plantarum* HY7714 at 10^9^ CFU per day for 8 weeks in hairless mice, decreased UV-induced epidermal thickness and suppressed the TEWL (Ra et al., 2014). Using an *in vitro* model, the differentiation and proliferation of keratinocytes was enhanced by means of a product composed by the plant *Scutellaria baicalensis* fermented with a strain of *L. plantarum* (Lee, 2019). A clinical trial with healthy volunteers supplemented with candies containing 2.1% *L. plantarum* lysates vs. candies not containing bacterial lysates for 8 weeks, showed a significant decrease in the TEWL and increase of skin hydration in face and forearm of the experimental-candy subjects (Kim H. et al., 2015). Other randomized, double-blind, placebo-controlled, clinical trial in healthy female volunteers revealed that treatment with heat-killed *Lactobacillus casei* subsp. *casei* 327 at 10^11^ CFU per day decreased the TEWL (Saito et al., 2017). Finally, drinking of *Lactobacillus helveticus*-fermented milk whey for 5 weeks significantly lowered the TEWL in hairless mice with sodium lauryl sulfate-induced dermatitis (Baba et al., 2010).

## 6 Conclusion

Rosacea is a multifactorial disease which causes a relevant deterioration in the quality of life of the patients. The pathophysiology of rosacea is becoming increasingly well understood, but the role of the skin and gut microbiota as well as certain bacteria and other specific microorganisms must be clarified. The impact of the gut-skin axis on rosacea has been little explored, in contrast to other inflammatory skin diseases such as atopic dermatitis or psoriasis. The clinical progression of patients with rosacea, despite the current available therapies approved by medicine agencies, is not always adequate and in frequent cases these patients will have a sustained symptomatology with frequent flare-ups. It is therefore imperative to explore more effective and safe treatments or therapeutic schedules for rosacea. Introduction and consolidation of new culture-independent techniques based on NGS of the *16S rRNA* gene in recent years has enabled to obtain unprecedented information about the microbiome. Studies characterizing the skin microbiota using this methodology in rosacea patients are scarce and recent. Similarity analyses of cutaneous microbiota between rosacea cases and healthy controls, or between affected and unaffected skin have provided contradictory results (Zaidi et al., 2018; Rainer et al., 2020; Wang et al., 2020; Woo et al., 2020; Thompson et al., 2021). Several differences between rosacea subtypes and controls have been detected at species level and these associations could be responsible for a relationship between rosacea and its comorbidities (Rainer et al., 2020; Thompson et al., 2020). Moreover, rosacea severity is related to changes in skin microbiota (Zaidi et al., 2018; Woo et al., 2020). On the other hand, studies characterizing the gut microbiota of rosacea patients based on NGS are also scarce. In this regard, significant differences have been consistently identified at genera level between rosacea patients and rosacea-free individuals (Nam et al., 2018; Chen et al., 2021; Moreno-Arrones et al., 2021). All these findings at skin and gut microbiota level reinforce the role of the skin-gut axis in the pathophysiology of rosacea. At this point and at this moment, oral probiotics, or even topical probiotics (mainly postbiotics) would come into play. However, we identify a deficiency of preclinical and human clinical trial evidence on the efficacy of these products in rosacea patients. In this narrative review we have established the basics and compiled the main directions of current knowledge to understand the mechanisms by which the microbiome influences the pathogenesis of rosacea, and how modulation of the skin and gut microbiota could benefit these patients.

## Author contributions

PS-P: Writing – original draft, Writing – review & editing. CE-M: Writing – review & editing. JG-G: Writing – review & editing. ML-V: Writing – review & editing. LN-M: Writing – review & editing. EN-D: Writing – review & editing. JA-S: Writing – review & editing. VN-L: Conceptualization, Writing – review & editing.
